# Self-perceived mental health and factors associated with the mental health of Hong Kong's asylum-seekers and refugees – A mixed methods study

**DOI:** 10.1016/j.heliyon.2023.e13481

**Published:** 2023-02-04

**Authors:** Isabella Ng, Joanne W.Y. Chung, Sharice F.Y. Choi, Vincent C.M. Yan

**Affiliations:** a Department of Asian and Policy Studies, The Education University of Hong Kong, Hong Kong; b School of Nursing and Health Studies, Hong Kong Metropolitan University, Hong Kong

**Keywords:** Mental health, Wellbeing, Asylum seekers, Refugees, Post-migration, Hong Kong

## Abstract

**Rationale:**

The 21st century has seen vast flows of displaced people. In the year 2020 alone, an estimated 11.2 million were forcibly displaced as a result of persecution, conflict or generalized violence. The torment and fear of war, persecution and threat to life, whether in the home country, during the process of fleeing, or in the post-migration host country, can be extremely traumatic to these marginalized populations. Hong Kong is not a signatory to the 1951 UN Refugee Convention, but the territory has signed the 1967 UN(CAT) which requires the former colony to allow people who flee for their lives to have their cases processed in Hong Kong. Currently there are around 14,000 cases in Hong Kong, some of whom have been in Hong Kong for more than a decade, waiting for their claims to be processed, living on meagre government subsidies and with no right to work.

**Objectives:**

The paper examines the mental health of asylum-seekers and refugees in Hong Kong and the factors associating with their mental health.

**Method:**

A sequential mixed methods approach was conducted among asylum-seekers and refugees in Hong Kong between October 2019 and mid-2020. It comprised a pilot quantitative survey conducted with 47 participants, and follow-up qualitative interviews with 16 of the 47 participants. Survey results were analyzed using statistical measures while the qualitative in-depth interviews were thematically analyzed to identify emergent patterns and categories.

**Results:**

Results from the quantitative data identify 52.2% of the asylum-seekers taking part as having symptomatic anxiety, 55.3% as having symptomatic depression and 54.3% as having overall problems. Qualitative results show that asylum-seekers and refugees cited lack of work and poverty as key factors affecting their mental health and well-being. Fear of being sent home was expressed by respondents who were married or having children for the fear of being separated from each other.

## Highlights

• Over half of the respondents have symptomatic depression and anxiety.
• Poverty is the main reason causing depression and anxiety.
• Not allowed to work is another factor causing depression and anxiety.
• Those who have families are afraid of being sent home for fear of separation.

## 1 Introduction

The study examines the mental health of asylum-seekers and refugees in Hong Kong and factors associating with their mental health in the post-migration state. In Hong Kong, there are around 14,000 non-refoulement claimants currently going through the process known as the Unified Screening Mechanism or USM (Immigration Department, personal communication, October 18, 2021), an assessment system that was introduced to replace the two-track system that existed before March 2014. Under the current USM, applicants’ claims are assessed against a wide array of possible risks including risk of persecution, risk of violation of the right to life (referring to Article 2, Section 8 of the Hong Kong Bill of Rights Ordinance Cap. 383, HKBOR), and risk of torture or cruel, inhuman or degrading treatment or punishment (referring to Article 3 of HKBOR). The process could lead either to the applicant being granted refugee status, or being deported back to their home country [1]. Many asylum-seekers and refugees (ASRs) who have been waiting to be processed have been in Hong Kong for many years, not permitted to work and thus solely reliant on in-kind government assistance [[2], [3], [4]]. A number of news media reports claim that some asylum-seekers and refugees have resided in Hong Kong for 10 years or longer [5,6].

Like any forcibly displaced migrants, the constant pressure facing the asylum-seekers and refugees in Hong Kong from the threats of deportation, poverty and discrimination may associate with their health and mental well-being. Wong et al. [7,8] have published two articles on African asylum-seekers in Hong Kong which highlight discrimination as one of the social determinants of access to health services. Using quantitative survey methods, the two papers provide interesting and invaluable insights on the African ASRs in Hong Kong. However, the focus of the studies is limited to this specific group and to examining the social determinants of well-being such as difficulties in accessing health services and living with or without family members in Hong Kong. There have been no studies published which look at the mental health status of asylum-seekers and refugees as a heterogeneous group in Hong Kong, where the majority of ASRs are from South Asian countries such as India, Pakistan and Bangladesh, and Southeast Asian countries such as the Philippines and Indonesia [9]. While the ASR population in Hong Kong is relatively small compared with other parts of the world, a study of the mental health status and the factors associating with the mental health of this marginalized group could provide invaluable insights into forced migration in East Asia, particularly if it includes an analysis of the processing time of claims and how this could be associated with the mental health of ASRs. In this context, it is important to note that Hong Kong is often only a transit point for the claimants; those who are recognized as refugees are usually sent to other countries, while the remainder are deported back to their own countries.

Since Hong Kong has not signed the UN's 1951 Refugee Convention, it has no obligation to house any recognized refugees. However, because the former British colony has been a signatory to the Convention against Torture and Other Cruel, Inhuman or Degrading Treatment or Punishment (hereafter UN(CAT)) since 1992, it does have an obligation not to expel or repatriate anyone who fears for their lives should they return to their home country. Under the terms of UN(CAT), signatories have to allow non-refoulement claimants to remain in Hong Kong while their cases are processed, and until the assessment is complete. As noted, claimants are not allowed to work, and must rely on the monthly payments provided by the International Social Service (ISS), a subcontractor of the government's Social Welfare Department. They receive HK$1500 housing allowance per adult, and HK$750 per child which will be transferred to the landlord directly every month, a HK$1200 food card, and money for transportation. The level of the assistance is pitched to prevent ASRs from becoming destitute but without creating a magnet effect [10]. In reality, however, living in one of the most expensive cities in the world and not being allowed to work, the meagre assistance from ISS is insufficient to prevent the non-refoulement claimants from becoming destitute [3,11]. The position of Hong Kong is unusual, since it is the only wealthy and cosmopolitan city or state that has not signed the 1951 Refugee Convention but is a signatory to the UN(CAT) convention. Its unique position allows ASRs to remain in transit in Hong Kong, awaiting the results of their claims. This makes it an interesting case to study how the asylum-seekers and refugees who must survive in this liminal state are affected in terms of their mental health and well-being.

Voluminous research has been conducted on refugees' mental health and factors associated with their psychological well-being in South America, Europe, the USA and Asia, including asylum-seekers and refugees in different situations [7,8,[12], [13], [14], [15], [16], [17], [18], [19], [20]]. Some focus on specific circumstances such as detention [19,21] or on refugee housing facilities [17]; some focus on particular issues like access to health services [7,8], the relationship between trauma and post-migration experiences [22], and the long years living in a host country [12,20]; others focus on pre-migration and/or post-migration experiences [15,23]. In Hong Kong, however, asylum-seekers and refugees are in a particularly transient position, expecting either to be deported back to their home countries should their cases fail, or to be accepted by third countries should their cases be upheld. Factors associated with ASRs’ mental health in Hong Kong could thus share common features with those identified in existing studies, but variations could also exist because of the transitory nature of the asylum-seekers and refugees in Hong Kong.

This study therefore aims to fill a gap by examining the factors associated with the mental health of a group of asylum-seekers and refugees in Hong Kong, who are of different origins and nationalities. The study looks into the post-migration experience and seeks to understand how these are associated with the mental health of ASRs when they are still in the in-between stage, unsure if they are able to stay but also unable to leave. The study adopts a mixed-methods approach, using a quantitative survey and qualitative in-depth interviews to probe the mental health status of asylum-seekers and refugee participants, as described in detail in the following section.

## 2 Method

### 2.1 Research design

This study is a preliminary investigation for a larger research project on mental health and rights to work of asylum-seekers and refugees in Hong Kong. The study consisted of two parts: a quantitative survey with interviewer-administered questionnaires targeting 50 refugees who have been in Hong Kong since claiming asylum. We included all ASRs who are 18 or above. Those who are below 18 are excluded in this study. They were divided into three groups: 1) those who had been in Hong Kong for three years or less; 2) for four to six years; or 3) for seven years or more. The questionnaire included questions on socio-demographic data, including gender, age, religion, duration of stay in Hong Kong and number of years in Hong Kong since applying for asylum; it also included questions taken from a range of existing tools which have been designed to assess the mental health status of respondents: the Hopkins Symptoms Checklist, the Post-Migration Living Difficulties Questionnaire, the Harvard Trauma Questionnaire and the Penn State Worry Questionnaire. More details of these tools are presented in Section 3.4 (below). In this paper, we focus on the discussion around mental health and the factors associated with the current mental health status of ASRs in Hong Kong. The design is based on previous studies on the ASR's group mental health and well-being [[24], [25], [26]].

### 2.2 Participants and study procedures

Since asylum-seekers and refugees are left to find their own accommodation in Hong Kong, rather than being confined in a camp or in particular kinds of houses, it is not easy to locate them. We work with two Non-governmental organizations (hereafter NGOs) that assist ASRs in Hong Kong, one located in the urban area and the other in a rural area. Details of the research and the contact number of the Principal Investigator (PI) were provided to the NGOs so as to allow direct contact between the participants and the PI and to ensure that participation in the study was entirely voluntary.

Recruitment of participants started in October 2019 and was completed in June 2020. Asylum-seekers and refugees were invited to participate in the study, with professional interpreters hired to conduct instant translations for participants who could not understand English. The interpreter is a paid individual from the community who had been trained in confidentiality and interview-specific issues and the interpreter has also been trained for this research. All English-speaking participants were interviewed by trained research assistants. A total of 47 participants were recruited from the two NGOs. The first element of the study was an administered face-to-face survey with trained research assistants explaining the research aims and procedures and the consent that was needed from the participants themselves. The quantitative survey was conducted in the university or the locations at the participants’ choice. When the questionnaire was complete, the participants were asked if they would be interested in having a follow-up in-depth interview. Each participant was given HKD$500 as an honorarium for their participation each time.

### 2.3 Ethical considerations

The study protocols were approved by the Human Research Ethics Committee (Approval Number: 2018-2019-0345). An information sheet explaining the study procedures, information about voluntary participation, explanation of risks that might occur, the right to withdraw, and the guarantees of anonymity and confidentially was made available to each participant, and written informed consent was obtained from each subject prior to the survey and interview.

### 2.4 Design of the questionnaire

A questionnaire with multiple sections was distributed to participants. Based on existing studies on the ASR [24,26] we design the questionnaire. The questionnaire started by collecting information about the participants: the first part dealt with basic demographic information, such as gender, date of birth, education level, ethnic group and asylum status, etc., while the second part comprised questions related to refugees and their personal circumstances, such as the number of family members they live with, and those left behind. The remainder of the questionnaire was based on five instruments used for measuring the post-migration difficulties, mental health status and traumatic events experienced by ASRs, as detailed below.

#### 2.4.1 Hopkins Symptoms Checklist – 25 (HSCL-25)

HSCL-25 is a self-reporting tool for evaluating symptoms of anxiety and depression. It comprises 25 items with responses on a 5-point Likert scale: “not at all”, “a little bit”, “moderately”, “quite a bit” and “extremely”. For our study, the answers were converted to score 0 to 4. The 25 items were divided into two dimensions: 10 items for measuring anxiety and 15 items for measuring depression. The anxiety score (mean of the 10 items), depression score (mean of the 15 items) and total score (mean of all items) were calculated. A score <1.75 was classified as “not symptomatic” and ≥1.75 was classified as “symptomatic”. As a tool, HSCL-25 has shown very good validity (0.89 for anxiety and 0.92 for depression) [27] and internal consistency (0.73–0.86) [28].

#### 2.4.2 Post-migration living difficulties (PMLD)

PMLD is a self-evaluation questionnaire for assessing unpleasant life experiences after migration. The questionnaire comprises 26 items that cover common problems in the areas of employment, isolation from family members, difficulties in applying for refugee status and in accessing health services and welfare support, as well as poverty, cultural alienation and boredom [25]. The answers again form a 5-point Likert scale: “no problem”, “a little problem”, “somewhat of a problem”, “a fairly big problem” and “serious problem”. The responses were converted to score 0 to 4 respectively. The results are reported in frequency and percentage for each item.

#### 2.4.3 Harvard Trauma Questionnaire (HTQ)

The HTQ is designed to identify past experiences of trauma events in war-affected communities and associated post-traumatic stress disorder [25]. HTQ includes three parts. Part 1 consists of 46 items which are considered common trauma experiences related to war-affected events, with the answer for each item being dichotomous (i.e. “yes” or “no”). The “yes” answers were reported in terms of frequency and percentage. Part 2 investigates experiences of brain injury through eight items. For items 1–6, the subjects need to answer “experienced?” (“yes” or “no”), “loss of consciousness?” (“yes” or “no”) and “if yes, for how long?” (expressed in hours and minutes). For item 7, the subject is asked to write down their normal weight and their starvation weight, while item 8 asks whether the subject was near to death, due to starvation; the answer is a dichotomous “yes” or “no”. “Yes” answers were reported as frequency and percentage and the numbers were reported as mean and standard deviation (SD). Part 3 looks at post-traumatic stress disorder (PTSD). This part comprises 40 items with answers on a 4-point Likert scale: “not at all”, “a little”, “quite a bit” and “extremely”. Amongst the 40 items, the mean of 16 items is the DSM-IV score^1^ and the mean of all items is the total score. In both cases, subjects are classified as “Symptomatic for PTSD” if the score is > 2.5.

#### 2.4.4 The Penn State Worry Questionnaire (PSWQ)

The PSWQ is a self-administered questionnaire designed to measure worry [29]. It contains 16 items with answers on a 5-point Likert scale, from 1, “not at all typical of me”, to 5, “very typical of me”. The total score was calculated and classified into one of three worry levels: low (16–39), moderate (40–59) or high worry (60–80). The concurrent validation showed good correlation of PSWQ (r = 0.40 to 0.74) with several existing anxiety inventories (e.g. State-Trait Anxiety Inventory and Beck Anxiety Inventory) when assessing anxiety, with an internal consistency of 0.86 [30].

#### 2.4.5 Activities to cope with life difficulties

This is a bespoke self-reporting questionnaire for identifying the activities undertaken by asylum-seekers and refugees to try to solve difficulties in their daily lives. The questionnaire consists of 14 items covering spiritual, social and distress-related behaviours [31,32]. The answers are on a 4-point Likert scale, including “none of the time”, “a little of the time”, “quite a lot of the time” and “most of the time”. The results are reported in frequency and percentage for each item.

### 2.5 Statistical analysis

SPSS 26 was used to process the data. The quantitative analysis was descriptive. The results of the demographic data and items in questionnaires were reported as frequency and percentage for ordinal and nominal variables and mean and standard deviation for numerical variables. Chi-square tests have been used to compare the differences of items or dimensions in all questionnaires between female and male [26]. And correlations have been used to look at possible associate factors to the ASR's mental health and it is strictly for descriptive purposes [[24], [25], [26]].

### 2.6 Qualitative in-depth interview

After conducting the quantitative questionnaire, all participants were invited for follow-up in-depth interviews. 16 out of the 47 participants participated in follow-up interviews in order to detailed understand their psychological distress and their wellbeing. Data on socio-demographic information, such as their background, household, and marital status, were collected during interviews. The interview questions were designed based on the quantitative survey results, including the history of the participants, the hardest part living in Hong Kong, and the supports obtain from the government and different organizations, and their assessment on the role of organizations. Face to face interviews of around 1 h were conducted by one of the authors between January and April 2020 at the location chosen by the participants to ensure that they felt safe and comfortable during the interview. Qualitative data from open-ended questions have been found to provide rich information from relatively few informants [33].

### 2.7 Qualitative data analysis

All the qualitative interviews were audio-recorded with consent of the participant and transcribed verbatim by the research assistants. Interview transcripts of the in-depth interviews were analyzed by thematic analysis [34]. The data analysis process of the interview transcripts involved coding material and identifying categories. These were analyzed using the framework approach. We identified several key themes which are relevant to the subject of this paper, without trying to fit the data into a pre-existing coding frame. Three coders were involved in the coding process in order to ensure inter-coder reliability. The data were analyzed by thematic analysis. Through identifying the major themes using thematic analysis, we then further identified the subthemes, for example in the hardest part living in Hong Kong, we had identified, (no)work, poor (poverty), (no) money, as the subthemes from the data we gathered.

## 3 Results from quantitative data

### 3.1 Participants

Forty-seven asylum seekers were recruited in this study: Table 1 presents relevant demographic data. There were 27 females (57.4%) and the average age of all participants was 40.8 years (SD 8.2 years). Ten of the subjects (21.3%) were Indonesian, making this the largest ethnic group in the sample. Thirty-nine subjects (84.7%) had graduated from secondary school or held higher qualifications. Twenty-four (51.1%) were Muslim. Six subjects (12.7%) were businessman in their country of origin, but none of them was allowed to work in Hong Kong. The average time spent in Hong Kong as non-refoulement claimants was 6.9 years (SD 3.5). The average time spent in Hong Kong before seeking asylum was 3.0 years (SD 2.7). A total of 28 (59.6%) subjects were married and were living in Hong Kong and, of these, 21 (75.0%) had one spouse only. However, amongst these married subjects, only 14 of them (50.0%) were living with their spouse. In terms of children, 16 subjects (34%) had two children. Twenty-four subjects (51.1%) had between one and three children living with them, one subject had five children but was not living with them. For the family member living in Hong Kong, the numbers of subjects have 0, 1 and 3 family member(s) were 12 (25.5%), and children is the family member that most subjects are living with (57.4%).

**Table 1:** Demographic information, asylum status and marital status of the participants (n = 47).

Demographics	Female		Male		Total	
	n	%	n	%	n	%
**Gender**	27	57.4	20	42.6	47	100.0
**Age**						
<20	0	0.0	0	0.0	0	0.0
20–29	1	3.7	4	20.0	5	10.6
30–39	12	44.4	5	25.0	14	36.2
40–49	12	44.4	6	30.0	17	38.3
>50	2	7.4	5	25.0	7	14.9
Mean ± SD (Years)	40.8 ± 1.5		40.8 ± 2.4		40.8 ± 8.2	
**Ethnic Group**						
Indonesian	9	33.3	1	5.0	10	21.3
Filipino	8	29.6	0	0.0	8	17.0
Bangladeshi	1	3.7	6	30.0	7	14.9
Egyptian	3	11.1	1	5.0	4	8.5
Indian	2	7.4	2	10.0	4	8.5
Pakistani	0	0.0	4	20.0	4	8.5
Nepalese	1	3.7	0	0.0	1	2.1
Others	3	11.1	6	30.0	9	19.1
**Highest Education Level**						
No Formal Education	2	7.7	2	10.0	4	8.7
Primary School	2	7.7	1	5.0	3	6.5
Secondary School	11	42.3	11	55.0	22	47.8
Vocational	4	15.4	0	0.0	4	8.7
University	6	23.1	5	25.0	11	23.9
Graduate/Professional	1	3.8	1	5.0	2	4.3
**Religious Affiliation**						
Muslim	12	44.4	12	60.0	24	51.1
Christian/Catholic/Orthodox/Pentecostal	13	48.1	5	25.0	18	38.3
No Religion	1	5.0	2	10.0	3	6.4
Jewish	1	3.7	0	0.0	1	2.1
Others	0	0.0	1	5.0	1	2.1
**Duration in HK as a Non-refoulement Claimant**						
0–5 years	15	55.6	11	55.0	26	55.3
6–10 years	10	37.0	4	20.0	14	29.8
11–15 years	2	7.4	5	25.0	7	14.9
Mean ± SD (Years)	7.2 ± 0.8		7.7 ± 1.5		6.9 ± 3.5	
**Duration in HK before Seeking Asylum**						
0–2 years	14	51.9	16	80.0	30	63.8
3–4 years	4	14.8	3	15.0	7	14.9
5–6 years	7	25.9	1	5.0	8	17.0
7–8 years	2	7.4	0	0.0	2	4.3
9–10 years	0	0.0	0	0.0	0	0.0
>10 years	0	0.0	0	0.0	0	0.0
Mean ± SD (Years)	4.0 ± 0.8		2.1 ± 0.7		3.0 ± 2.7	
**Permission to Work in HK**						
No	27	100.0	20	100.0	47	100.0
Yes	0	0.0	0	0.0	0	0.0
**Marital Status**						
Single (never been married)	4	14.8	3	15.0	7	14.9
Married	14	51.9	14	70.0	28	59.6
Divorced	4	14.8	3	15.0	7	14.9
Separated	5	18.5	0	0.0	5	10.6

*Percentages do not always add up to 100 because some subjects declined to answer.

### 3.2 Hopkins Symptoms Checklist – 25 (HSCL-25)

According to the results from the HSCL-25 part of the study, 52.2% of the asylum-seekers were identified as symptomatic with anxiety, 55.3% were identified as symptomatic with depression and 54.3% have overall problems. No significant difference was found between gender (Table 2).

**Table 2:** Symptomatic in anxiety and depression according to HSCL-25.

Score^#^		Female		Male		Total			X^2^	p*
		n	%	n	%	n	%			
**Anxiety Score (10 Items) (n** = **46)**										
	Not symptomatic (Score <1.75)	13	50.0	9	45.0		22	47.8	0.113	0.736
	Symptomatic (Score≥1.75)	13	50.0	11	55.0		24	52.2		
**Depression Score (15 Items)**										
	Not symptomatic (Score <1.75)	13	48.1	8	40.0		21	44.7	0.309	0.579
	Symptomatic (Score≥1.75)	14	51.9	12	60.0		26	55.3		
**Total Score (25 Items) (n** = **46)**										
	Not symptomatic (Score <1.75)	13	50.0	8	40.0		21	45.7	0.456	0.500
	Symptomatic (Score≥1.75)	13	50.0	12	60.0		25	54.3		

*Considered statistically significant when p ≦ 0.05.

#For each score, n = 47 unless specified.

### 3.3 Post-migration living difficulties (PMLD)

The numbers and percentages of asylum-seekers who reported “a fairly big problem” or “serious problem” among the items listed in the PMLD are shown in Table 3. The top ten post-migration living difficulties encountered by the asylum-seekers were:

1. Fears of being sent home (95.7%)
2. Returning home to family in an emergency (91.5%)
3. Poverty (not having enough money for basic needs) (89.4%)
4. Finding work (87.2%)
5. Worries about family back home (83.0%)
6. Separation from family (80.9%)
7. Loneliness and boredom (70.2%)
8. Isolation (loneliness, being or feeling alone) (63.8%)
9. Interviews by immigration department (61.7%)
10. Time taken in processing refugee/immigrant applications (59.6%)

**Table 3:** Post-migration living difficulties experienced by asylum-seekers in Hong Kong.

Question^#^	Female		Male		Total		X^2^	p*
	n	%	n	%	n	%		
Getting treatment for health problems	10	37.0	5	25.0	15	31.9	0.766	0.381
Access to emergency medical care (n = 46)	6	22.2	6	31.6	12	26.1	0.506	0.477
Access to long term medical care (family doctor, Primary Care Physician) (n = 44)	6	23.1	8	44.4	14	31.8	2.239	0.135
Access to dental care (n = 43)	11	44.0	13	72.2	24	55.8	3.380	0.066
Access to counselling services (if you wanted counselling, would it be problem for you?) (n = 44)	8	29.6	7	41.2	15	34.1	0.619	0.431
Government help with welfare (unemployment benefits, financial help)	11	40.7	11	55.0	22	46.8	0.938	0.333
Help with welfare from NGOs (n = 45)	9	33.3	8	44.4	17	37.8	0.567	0.451
Help with welfare from churches (n = 41)	4	16.0	7	43.8	11	26.8	3.827	0.050*
Time taken in processing refugee/immigrant applications	12	44.4	16	80.0	28	59.6	6.031	0.014*
Communication difficulties/Language difficulties	12	44.4	10	50.0	22	46.8	0.142	0.706
Discrimination	13	48.1	12	60.0	25	53.2	0.648	0.421
Finding work	23	85.2	18	90.0	41	87.2	0.239	0.625
Working conditions	–	–	–	–	–	–	–	–
Poverty (not having enough money for basic needs — food, clothing, shelter)	23	85.2	19	95.0	42	89.4	1.164	0.281
Separation from family	21	77.8	17	85.0	38	80.9	0.387	0.534
Worries about family back home	21	77.8	18	90.0	39	83.0	1.215	0.270
Return home to family in an emergency	25	92.6	18	90.0	43	91.5	0.099	0.753
Loneliness and boredom	17	63.0	16	80.0	33	70.2	1.595	0.207
Isolation (loneliness, being or feeling alone)	14	51.9	16	80.0	30	63.8	3.943	0.047*
Access to traditional foods	13	48.1	13	65.0	26	55.3	1.320	0.251
Interviews by immigration	17	63.0	12	60.0	29	61.7	0.043	0.836
Conflict with immigration officers	12	44.4	9	45.0	21	44.7	0.001	0.970
Fears of being sent home	27	100.0	18	90.0	45	95.7	2.820	0.093
Practising your religion	4	14.8	2	10.0	6	12.8	0.239	0.625
Adjusting to the weather/climate	7	25.9	2	10.0	9	19.1	1.882	0.170
Permission to work	–	–	–	–	–	–	–	–

*Considered statistically significant when p ≦ 0.05.

#For each question, n = 47 unless specified.

As no asylum-seekers are allowed to work in Hong Kong, the answers to the questions “working conditions” and “permission to work” are left blank. According to the results of a Chi-square test, the questions “help with welfare from churches” (X^2^ = 3.827, p = 0.050), “time taken in processing refugee/immigrant applications” (X^2^ = 6.031, p = 0.014) and “isolation (loneliness, being or feeling alone)” (X^2^ = 3.943, p = 0.047) were significantly associated with gender (Table 3).

### 3.4 Harvard Trauma Questionnaire (HTQ)

The percentage of asylum-seekers who were classified as symptomatic for PTSD according to the HTQ Part 3 is shown in Table 4. The DSM-IV score revealed that 42.6% of the asylum-seekers were symptomatic for PTSD. The total score showed 40.4% of the asylum-seekers were symptomatic for PTSD. A Chi-square test suggested that there was no correlation between gender and PTSD; however, this may be because the sampling was too small.

**Table 4:** Number and percentage of asylum-seekers classified as symptomatic for PTSD according to HTQ Part 3 – Trauma Symptoms (n = 47).

Score		Female		Male		Total		X^2^	p*
		n	%	n	%	n	%		
**DSM-IV Score (16 Items)**									
	Not symptomatic for PTSD (Score≤2.5)	18	66.7	9	45.0	27	57.4	2.206	0.137
	Symptomatic for PTSD (Score >2.5)	9	33.3	11	55.0	20	42.6		
**Total Score (40 Items)**									
	Not symptomatic for PTSD (Score≤2.5)	18	66.7	10	50.0	28	59.6	1.325	0.250
	Symptomatic for PTSD (Score >2.5)	9	33.3	10	50.0	19	40.4		

*Considered statistically significant when p ≦ 0.05.

### 3.5 The Penn State Worry Questionnaire (PSWQ)

According to this section of the study, 38.1% of the asylum-seekers had a high worry level, 45.2% had a moderate worry level and 16.7% had a low worry level (Table 5). The Chi-square test revealed that there was no association between gender and the classification of worry levels.

**Table 5:** Number and percentage of asylum-seekers classified as low worry, moderate worry and high worry according to the PSWQ (n = 42).

Classification	Female		Male		Total		X^2^	p*
	n	%	n	%	n	%		
Low Worry (16–39)	5	20.8	2	11.1	7	16.7	0.420	0.811
Moderate Worry (40–59)	12	50.0	7	38.9	19	45.2		
High Worry (60–80)	7	29.2	9	50.0	16	38.1		

*Considered statistically significant when p ≦ 0.05.

Correlation was performed to examine the association between poverty and HSCL-25 which is a self-reporting tool for evaluating symptoms of anxiety and depression, and the PSWQ which measure worry (Table 6). Poverty has shown to be significantly positively correlated with the HSCL-25 total score, HSCL anxiety score and HSCL Depression score, and PSWQ.

**Table 6:** Relationship between poverty and HSCL and PSWQ.

		HSCL Total Score	HSCL Anxiety Score	HSCL Depression Score	PSWQ Total Score
PMLD_Q14. Poverty (not having enough money for basic needs--- food, clothing, shelter)	Pearson Correlation	.449**	.333*	.482**	.433**
	Sig. (2-tailed)	.002	.024	<.001	.003
	N	46	46	47	46

**Correlation is significant at the 0.01 level (2-tailed).

*Correlation is significant at the 0.05 level (2-tailed).

## 4 Results from qualitative data

Among the 16 participants, there are 12 females and four males. Seven out of 16 participants come from Indonesia, four from the Philippines, two from Bangladesh, and one each from Kazakhstan, Pakistan, and Uganda. Most (11 out of 16) of the participants are married, four separated and one divorced. A total of 14 participants have children living together in Hong Kong, while one participant does not have a child and one participant revealed that his children are living are living together with him. In terms of the duration in Hong Kong as a non-refoulement claimants, eight participants have been in Hong Kong as non-refoulements for less than five years, five participants for six to ten years, and three have become non-refoulement claimants for over ten years. All the 16 participants are not allowed to work in Hong Kong.

Results from the qualitative data are consistent with the findings from the survey regarding the mental health and well-being of asylum-seekers and refugees in Hong Kong. Half of the respondents expressed that they were “stressed” and “finding life extremely difficult” with some mentioning that they “had headaches” and “cannot sleep”; one respondent told us that he “has been thinking a lot”, and even thought people would comment that he “looks very old at his age” because of the stress. This section details some of the main factors associating with the mental health and well-being of ASRs in Hong Kong, as revealed by the follow-up interviews.

We analyzed the interviews in terms of the factors associated to the mental health of asylum-seekers and refugees using thematic analysis, and three recuring themes were identified: (1) poverty; (2) fear of being sent home; (3) lack of right to work (Table 7).

**Table 7:** Themes and subthemes in the qualitative analysis.

Theme	Subtheme	Number of participants
Poverty	Not having enough money to meet basic needs	16
	Not having enough money to buy things for children	9
	Not enough subsidies	12
	Borrow money	7
Fear of being sent home	Separation of the family	2
	Possibility of facing threats	4
Lack of right to work	No money to support daily needs	15

Consistent with the findings in the quantitative survey, all the interview respondents reported that poverty was the major factor contributing to stress, telling us about the stress and depression that came from not having enough money to make ends meet. Janice,^2^ a 39-year-old Filipina claimant who has been in Hong Kong since 2004, was finding that not having work and not having enough money to meet her basic needs and to buy things for her child was stressing her out.

The expensive house rent, electricity and water; ISS doesn't increase the money for the house rent. The food is still okay. But the rent is very difficult because it's so expensive. Not only that one. You need to buy things for your daughter too. Very hard because you don't have a job.

Ben and Amy, a Bangladeshi/Indonesian couple who have been in Hong Kong as non-refoulement claimants for over 10 years, reported that they could not sleep, and trying to meet the demands that come with having kids was a real headache because they could not afford it.

Yanny, who sought asylum in Hong Kong in 2014, told us that she and her husband, who is also a non-refoulement claimant, are stressed and upset about not having enough money to support the daily lives of the family and buy food for their baby. Moreover, they fight because of the financial difficulties they face.

Because I have two babies … the government doesn't pay enough. The rent is expensive. What the government pays is not enough for us. For food, it's also not enough for us. […] Yeah, sometimes [we are] stressed and upset. We, the family, sometimes fight about that. Always fight about that. My husband not working. Me not working. And we don't have any money. Sometimes when the baby's food is gone, the baby's milk is gone, we always fight about that.

A similar response came from Ivy, who became non-refoulement claimant since 2014, was consistently under stress because she could not provide for her kids, for instance buying toys.

Sometimes stress, sometimes what is it called? Or my temperature is high — because of stress, thinking too much. Not have money and then the kids want this one, sometimes want buy toys, I cannot buy.

Another issue that respondents identified as a source of stress is not having the right to work in Hong Kong. Having paid work is key component for them to solve their financial problems. Shirley, a 39-year-old Filipina who has been a non-refoulement claimant for nine years, expressed it as follows:

For me, you know madam, freedom is a big part of your life, right? Being a refugee, we cannot work. We cannot do anything. It’s like you don't have wings, right? To earn your own money. So if they just give me the freedom to do something, even if we have a child, maybe there is a lot of work that we can do. When they go to school, we can have some part-time jobs, right? So after we can earn our own money, right? Not just stay like this. When this money is finished, what can we do? The stress will come. Ahh, I don’t have money anymore.

Mandy, a Filipina who has also been a non-refoulement claimant in Hong Kong for nine years, expressed her desperation at not being able to work and shared Shirley's view that having the right to work would allow her to better take care of her children.

I'm shy to beg anyone, I always say we're okay, no problem. But actually, it's very difficult. When I was working, I had money, I never asked anyone but now you're begging, the [other] person can't understand your situation. Yes, I'm strong, I can do work. I can do work if they allow me to work. I will work but I have four children, I don't want to take the risk to work. [What if] something happens to me. How about my children? If I can, I don't want to choose to be an asylum seeker. It's very hard in HK.

Six out of the 16 participants mentioned that the threat of being sent home also caused them worry and stress. They were haunted by a fear of being sent home and separated from their current family in Hong Kong, and even the possibility of facing death threats because their children were born out of wedlock. Polly, a Filipina who has been in Hong Kong as a non-refoulement claimant since 2017, told of her fear that she will be sent back to the Philippines and her worry that she will not be protected there.

It's my fear that they will deport me to go back to there. But it's something that I'm trying to confront also, because it's very difficult to live in this kind of situation. Although I know that Justice Center is kind and helpful to push my case, but it would still be different. It would be different if I gained back my confidence and was able to be protected by my country.

Nani had similar worries:

And I’m worried when I go back. My son has no father. And I’m not married. Indonesia [is] Muslim. I’m worried. But now my life is better because I have my son. I’m happy. Before I was overthinking. I cried.

Couples who are of different nationalities and who met and married in Hong Kong indicated that they were worried about being sent home because this would imply separation from each other. Ben and Amy, the Bangladeshi/Indonesian couple mentioned above, met and married in Hong Kong, having lodged their claims when they were still single. They told us that they are “just worried that (their) cases will be finished and (they) cannot be together again”.

Sarah, a Filipina who has become a non-refoulement claimant in Hong Kong since 2004, shared her anxieties:

Yeah. Exactly. It’s a lot of fear (to be sent home). It’s a daily nightmare for anything. You cannot move. Among our friends, it is the same situation. So it’s all in one type of the situation. It’s not easy to come out. The person like me, an asylum seeker. It isn’t easy.

Less than half of the interview participants identified a fear of being sent home, which is not consistent with our quantitative findings. However, interview responses on what issues are considered to be the hardest part of living in Hong Kong were consistent with the quantitative findings. For example, all of them expressed their fear of living under poverty. They expressed their anguish at not having enough money to live day by day, and worried about what would happen if their children were in trouble, for instance having health issues and needing to go to hospitals.

The lack of right to work was another issue that the respondents found particularly hard; this is consistent with the quantitative results which show that finding work is the most difficult (because they do not have the right to work). This is closely related to poverty in their daily lives, with all the respondents pointing to the extremely high cost of renting in Hong Kong, and the fact that government subsidies are not enough to support them. They therefore need to borrow money from their friends, or get extra support from churches or NGOs. In the discussion about government support for ASRs in Hong Kong, there was consensus among the respondents that the Hong Kong government has not been giving adequate support to them; coupled with the lack of a right to work, their lives in Hong Kong are therefore extremely difficult.

## 5 Discussion

Using a mixed-methods approach of quantitative survey and follow-up qualitative interviews, this study examined the post-migration experiences of asylum-seekers and refugees in Hong Kong, with a focus on their mental health and quality of life. The study was conducted with the aim of advancing the understanding of the mental health of this marginalized group, who has been understudied on their well-being aspect. The project looks into the post-migration experience and seeks to understand how these are associated with the mental health of ASRs when they are still in the in-between stage, unsure if they are able to stay but also unable to leave. In this study, we examined 47 participants by quantitative survey and subsequently 16 interviewees for interviews. We contributed to the understanding factors associated with the mental health of a group of asylum-seekers and refugees in Hong Kong, who are of different origins and nationalities.

The quantitative results in the pilot test suggest half of the respondents have symptoms of anxiety and depression. Our descriptive findings showed that 95.7% of the respondents had the fear of being sent home, followed by returning home to family in an emergency as a big or serious problem; followed by 89.4% finding poverty (not having enough money for basic needs) and finding work (87.4%) as major and or serious problem. We would like to understand in details how this affects their lives from qualitative in-depth interviews.

With reference to the pilot study findings, in our qualitative interviews, we further investigated the ASRs’ well-being on several key issues: 1) the challenges and difficulties they face in life and how that impact their mental health and their daily activities; 2) their relationship with their original home and their feelings of staying in Hong Kong, with the possibility that they might be sent home at any given point. Results from the qualitative findings show that respondents are feeling “stressed and depressed” because of not having right to work, a key factor contributing to their poverty.

All the respondents expressed the hardest part living in Hong Kong is that they do not have enough money for a living; and government did not provide enough subsidies to support them. What made their lives more difficult and stressful was that they could not work. That implied that they did not have the flexibility of spending on essential items for their children should they need help; and the families for basic necessities because everything was circumscribed by the government. The findings correspond to studies by Refs. [12,15,22]. All these studies found that (lack of) and poverty are powerful determinants for ASRs’ mental health.

### 5.1 Self-perceived mental health, poverty and lack of right to work

The quantitative pilot results show that respondents are facing stress and showing symptoms of anxiety and depression, with the reasons for stress mainly associated with poverty in the post-migration period (that is, living in Hong Kong), and the lack of right to work — a major factor that contributes to the symptoms of anxiety and depression in the group. Further analysis using correlations also show a significant correlation between poverty and HSCL-25 and PSWQ. This corresponds to previous studies [22,35] suggesting that poverty is one of the major determinants of ASRs’ mental health and well-being. Qualitative results not only show that the entire respondents population found that “not having enough money to meet basic needs” and “not enough subsidies” are causing their daily stress and worries; the lack of work rights which caused them not having “money to support daily needs” also emerged as the key theme as the biggest challenges. The problems they are facing daily are rooted on financial problems and the lack of viable options to get money legally. The result resonate previous studies by Refs. [12,15] on the importance of income and right to work.

### 5.2 Fear of being sent home

In the qualitative in-depth interviews, almost half of the respondents indicated that they were worried about being sent home and that this caused them insomnia and stress, as suggested in the initial quantitative pilot study. Other studies have identified fear of being sent home was one of the major factors in causing mental health issues [22,36,37] In our study, we found that respondents who expressed their fear of being sent home in the qualitative interviews were most likely to be married or to have children. Married couples are afraid of separation from their spouses in the event that their cases have different outcomes, while single parents with children born out of wedlock fear for the safety of their children should they be returned to their home countries.

The results on symptomatic anxiety and symptomatic depression in this study correspond to previous studies on trauma and post-migration experience [22,38].

However, there are some results emerged that are beyond our expectation. For example, unlike other studies which looked at how long years of waiting is one of factors associated with their mental health and well-being [12,20], our study did not show any significant relationship between the two. From the qualitative results, all the participants who has been in Hong Kong as non-refoulement claimants for less than 5 years, like Yanny and Polly, or longer period of time, like Sarah, Ben, and Amy, revealed that they feel “stressed” and “finding life extremely difficult” due to poverty and lack of right to work. This might be because the ASRs feel safe in the host country; therefore, the years of waiting for a decision do not impact them as much as the fear of being sent home.

With some of the interesting discrepancies from previous studies, it is therefore, imperative for researchers to further study on the entire ASR population in Hong Kong to understand in detail the mental health and well-being of the ASRs and the associated factors to it.

### 5.3 Other findings: differences on post-migration anxiety, depression, worry and living difficulties between gender

According to the quantitative pilot test results from the used assessment tools, no difference was found in HSCL-25 (measuring anxiety and depression), HTQ – Part 3 (measuring PTSD) and PSWQ (measuring worry level) between female and male. However, from the PMLD, male showed that they have significantly more living difficulties when seeking help/welfare from NGOs and churches than female; meanwhile, male revealed they have significantly stronger feeling in isolation than female as well. While we followed this reference to see whether there would be the gender difference in seeking help/welfare from NGOs, in the qualitative study, however, we did not see s significant difference in terms of gender on the relationship between PMLD and seeking help/welfare from NGO and churches and feeling in isolation. It may be because that the sampling is too small. A further investigation could be done on looking at gender difference regarding those variables with larger samples.

## 6 Strength and limitations

The study is a first attempt to conduct a sequential mixed-method study, with quantitative pilot study as reference to the follow-up qualitative in-depth interviews, to understand the mental health of a group of asylum-seekers and refugees in Hong Kong, who are of different origins and nationalities. The study looks into the post-migration experience and seeks to understand how these are associated with the mental health of ASRs when they are still in the in-between stage, unsure if they are able to stay but also unable to leave. The results of the study could help inform for future larger scale study on the group.

There are several limitations to this study. It relied on self-ratings and did not include a clinical examination in which information from several sources could be triangulated in order to assess the actual level of psychopathology. The analysis is also descriptive so it not generalizable. It is a preliminary study so the scope of participation was limited. Also, the participants were mainly from two major refugee NGOs in Hong Kong, even though the composition of the informants does give a sense of the variety of the asylum-seeking and refugee population in the territory. It would need to expand the research into a wider asylum-seeking and refugee community so as to better understand the overall health and well-being situation of the group.

## 7 Conclusion

This study examines the psychological well-being of asylum-seekers and refugees in Hong Kong and the factors associating with their mental health. Both the quantitative data and the qualitative results suggest that the participants of this research face immense stress, brought about by living as asylum-seekers and refugees in Hong Kong in the post-migration stage. Over half of the participants are experiencing symptomatic anxiety and/or depression. Although asylum-seekers and refugees receive monthly assistance from the government, both the quantitative and qualitative results of the study demonstrate that ASRs in Hong Kong are experiencing poverty and that this may adversely affect their quality of life and mental health. Moreover, from the qualitative interviews, the study revealed that poverty, lack of right to work and fear of being sent home are the most frequently cited factors underlying mental health difficulties. These findings are consistent with the questionnaire results. While the study is in no way representative, it is an indicator to suggest a more comprehensive study on the group is needed. The findings also indicate, if in no way strongly reflect that intervention should focus on managing ASRs' fear of being sent home, and on formulating policies which improve the situation of the group by granting them perhaps conditional right to work and relieving their poverty by providing more subsidies in the forms of cash. Further research such be conducted on what interventions asylum seekers themselves would like to see. We also need to look into more effective mental health treatment and social support for this vulnerable group, as well as improving our understanding of the impact of government policy on ASRs’ quality of life and well-being.

## Author contribution statement

Isabella Ng: Conceived and designed the experiments; Performed the experiments; Analyzed and interpreted the data; Contributed reagents, materials, analysis tools or data; Wrote the paper.

Joanne Waiyee Chung: Conceived and designed the experiments; Analyzed and interpreted the data; Contributed reagents, materials, analysis tools or data; Wrote the paper.

Sharice Fungyee Choi: Performed the experiments; Analyzed and interpreted the data; Contributed reagents, materials, analysis tools or data; Wrote the paper.

Vincent Chun Many Yan: Analyzed and interpreted the data; Contributed reagents, materials, analysis tools or data; Wrote the paper.

## Funding statement

This work was supported by the Faculty of Liberal Arts and Social Sciences, The 10.13039/501100010410Education University of Hong Kong [IDS-9/6th round].

## Data availability statement

Data included in article/supp. material/referenced in article.

## Declaration of interest's statement

The authors declare no conflict of interest.

## Footnotes

1 DSM-IV codes are the classifications used in the Diagnostic and Statistical Manual of Mental Disorders, known as DSM-IV-TR, published by the American Psychiatric Association. DSM-IV codes are used by mental health professionals to assess and identify mental disorders.

2 All names used in this paper are pseudonyms.
