# Supplementary material for: Self-perceived mental health and factors associated with the mental health of Hong Kong's asylum-seekers and refugees – A mixed methods study
Source: Heliyon. 2023 Feb 4;9(2):e13481. doi: 10.1016/j.heliyon.2023.e13481 (PMC9939599; doi:10.1016/j.heliyon.2023.e13481)
Supplement: Multimedia component 1 [file mmc1.docx]

The mental health of asylum-seekers and refugees in Hong Kong and factors affecting their mental health in the post-migration state – A mixed methods study

**Demographic Characteristics**

We would first like to get some general information about you.

1. What is your gender?

______ male

______ female

1. How old were you at your last birthday?

_________________ (please give your age)

1. What is the highest level of school you attended?

_____________ primary

_____________ secondary

_____________ vocational (secretarial, electrical, accounting)

_____________ university

_____________ graduate/professional

_____________ no formal education

How long have you been in Hong Kong as a non-refoulement claimant?

______________ (please give number of years)

1. How long have you been in Hong Kong before seeking asylum?

______________ (please give number of years)

1. What was your occupation in your country of origin (if from a country other than Hong Kong)?

__________________ (please give your occupation)

1. Do you get the permission to work in Hong Kong?

________ yes

________ no

1. To which ethnic group do you belong? (country from)

___________________ Pakistani

___________________ Indian

___________________ Filipino

___________________ Indonesian

___________________ Egyptian

___________________ Thai

___________________ Bangladeshi

___________________ Nepalese

___________________ Others, please specify: _________________________

1. What is your marital status?

_______ married (please also specify number of wives you have or that your husband has) _____ (number of wives) ______ (number of wives living with you)

______ single (never been married)

______ divorced

______ separated

______ widowed

______ engaged

1. How many children do you have?

____________ (please give TOTAL number of children)

How many children do you have that are living with you?

____________ (please give number)

How many children do you have that are NOT living with you?

___________ (please give number)

1. How many of your family members are living in Hong Kong?

__________ (please give number)

11a. Please list family members who are living in Hong Kong.

______ spouse

______ children

______ friends

______ parents

______ siblings

______ cousin

______ others, please specify _______________________________________

1. Who do you live with now?

______ spouse

______ children

______ friends

______ parents

______ siblings

______ cousin

______ others, please specify _______________________________________

1. What is your religious affiliation?

______ Christian/Catholic/Orthodox

______ Jewish

______ Islam/Muslim

______ Hindu

______ Buddhist

______Other, please specify_______________________________

**Post-Migration Living Difficulties (PMLD)**

We would like to ask you about some difficulties you may have experienced seeking asylum in Hong Kong. Please answer the questions using the following scale, and choose the response which is most appropriate for you.

0= No problem

1= A little problem

2= Somewhat of a problem

3= A fairly big problem

4= Serious problem

| 1. **Getting treatment for health problems** | | | | |
| --- | --- | --- | --- | --- |
| 0  No problem | 1  A little problem | 2  Somewhat of a problem | 3  A fairly big problem | 4  Serious problem |
| 1. **Access to emergency medical care** | | | | |
| 0  No problem | 1  A little problem | 2  Somewhat of a problem | 3  A fairly big problem | 4  Serious problem |
| 1. **Access to long term medical care (family doctor, Primary Care Physician)** | | | | |
| 0  No problem | 1  A little problem | 2  Somewhat of a problem | 3  A fairly big problem | 4  Serious problem |
| 1. **Access to dental care** | | | | |
| 0  No problem | 1  A little problem | 2  Somewhat of a problem | 3  A fairly big problem | 4  Serious problem |
| 1. **Access to counseling services (if you wanted counseling, would it be problem for you?)** | | | | |
| 0  No problem | 1  A little problem | 2  Somewhat of a problem | 3  A fairly big problem | 4  Serious problem |
| 1. **Government help with welfare (unemployment benefits, financial help)** | | | | |
| 0  No problem | 1  A little problem | 2  Somewhat of a problem | 3  A fairly big problem | 4  Serious problem |
| 1. **Help with welfare from NGOs (social services, e.g., Justice Centre Hong Kong, Hong Kong Society for Asylum-seekers and Refugees, Red Cross, Salvation Army)** | | | | |
| 0  No problem | 1  A little problem | 2  Somewhat of a problem | 3  A fairly big problem | 4  Serious problem |
| 1. **Help with welfare from churches** | | | | |
| 0  No problem | 1  A little problem | 2  Somewhat of a problem | 3  A fairly big problem | 4  Serious problem |
| 1. **Time taken in processing refugee/immigrant applications** | | | | |
| 0  No problem | 1  A little problem | 2  Somewhat of a problem | 3  A fairly big problem | 4  Serious problem |
| 1. **Communication difficulties/Language difficulties** | | | | |
| 0  No problem | 1  A little problem | 2  Somewhat of a problem | 3  A fairly big problem | 4  Serious problem |
| 1. **Discrimination** | | | | |
| 0  No problem | 1  A little problem | 2  Somewhat of a problem | 3  A fairly big problem | 4  Serious problem |
| 1. **. Find work** | | | | |
| 0  No problem | 1  A little problem | 2  Somewhat of a problem | 3  A fairly big problem | 4  Serious problem |
| 1. **Working conditions**   **□ Not working. Go to Question 14.** | | | | |
| 0  No problem | 1  A little problem | 2  Somewhat of a problem | 3  A fairly big problem | 4  Serious problem |
| 1. **Poverty (not having enough money for basic needs--- food, clothing, shelter)** | | | | |
| 0  No problem | 1  A little problem | 2  Somewhat of a problem | 3  A fairly big problem | 4  Serious problem |
| 1. **Separation from family** | | | | |
| 0  No problem | 1  A little problem | 2  Somewhat of a problem | 3  A fairly big problem | 4  Serious problem |
| 1. **Worries about family back home** | | | | |
| 0  No problem | 1  A little problem | 2  Somewhat of a problem | 3  A fairly big problem | 4  Serious problem |
| 1. **Return home to family in an emergency** | | | | |
| 0  No problem | 1  A little problem | 2  Somewhat of a problem | 3  A fairly big problem | 4  Serious problem |
| 1. **Loneliness and boredom** | | | | |
| 0  No problem | 1  A little problem | 2  Somewhat of a problem | 3  A fairly big problem | 4  Serious problem |
| 1. **Isolation (loneliness, being or feeling alone)** | | | | |
| 0  No problem | 1  A little problem | 2  Somewhat of a problem | 3  A fairly big problem | 4  Serious problem |
| 1. **Access to traditional foods** | | | | |
| 0  No problem | 1  A little problem | 2  Somewhat of a problem | 3  A fairly big problem | 4  Serious problem |
| 1. **Interviews by immigration** | | | | |
| 0  No problem | 1  A little problem | 2  Somewhat of a problem | 3  A fairly big problem | 4  Serious problem |
| 1. **Conflict with immigration officers** | | | | |
| 0  No problem | 1  A little problem | 2  Somewhat of a problem | 3  A fairly big problem | 4  Serious problem |
| 1. **Fears of being sent home** | | | | |
| 0  No problem | 1  A little problem | 2  Somewhat of a problem | 3  A fairly big problem | 4  Serious problem |
| 1. **Practice your religion** | | | | |
| 0  No problem | 1  A little problem | 2  Somewhat of a problem | 3  A fairly big problem | 4  Serious problem |
| 1. **Adjusting to the weather/climate** | | | | |
| 0  No problem | 1  A little problem | 2  Somewhat of a problem | 3  A fairly big problem | 4  Serious problem |
| 1. **Permission to work**   **□ No permit to work. Go to next questionnaire.** | | | | |
| 0  No problem | 1  A little problem | 2  Somewhat of a problem | 3  A fairly big problem | 4  Serious problem |

**Hopkins Symptom Checklist-25 (HSCL-25)**

Directions:

The following is a list of problems people sometimes have. Please read each one carefully, and choose the option that best describes HOW MUCH THAT PROBLEM HAS DISTRESSED OR BOTHERED YOU DURING THE PAST 7 DAYS, INCLUDING TODAY.

The options are as follows:

**0= NOT AT ALL (NA)**

**1= A LITTLE BIT (LB)**

**2= MODERATELY (MO)**

**3= QUITE A BIT (QB)**

**4= EXTREMELY (EX)**

In the past 7 days, how much were you distressed by…?

|  | NA | LB | MO | QB | EX |
| --- | --- | --- | --- | --- | --- |
| 1. Headaches | 0 | 1 | 2 | 3 | 4 |
| 1. Nervousness or shakiness inside | 0 | 1 | 2 | 3 | 4 |
| 1. Faintness, dizziness or weakness | 0 | 1 | 2 | 3 | 4 |
| 1. Loss of sexual interest or pleasure | 0 | 1 | 2 | 3 | 4 |
| 1. Feeling low in energy or slowed down | 0 | 1 | 2 | 3 | 4 |
| 1. Thoughts of ending your life | 0 | 1 | 2 | 3 | 4 |
| 1. Trembling | 0 | 1 | 2 | 3 | 4 |
| 1. Poor appetite | 0 | 1 | 2 | 3 | 4 |
| 1. Crying easily | 0 | 1 | 2 | 3 | 4 |
| 1. Feelings of being trapped or caught | 0 | 1 | 2 | 3 | 4 |
| 1. Suddenly scared for no reason | 0 | 1 | 2 | 3 | 4 |
| 1. Blaming yourself for things | 0 | 1 | 2 | 3 | 4 |
| 1. Feeling lonely | 0 | 1 | 2 | 3 | 4 |
| 1. Feeling blue (sad) | 0 | 1 | 2 | 3 | 4 |
| 1. Worrying too much about things | 0 | 1 | 2 | 3 | 4 |
| 1. Feeling fearful | 0 | 1 | 2 | 3 | 4 |
| 1. Heart pounding or racing | 0 | 1 | 2 | 3 | 4 |
| 1. Difficulty falling asleep and sleeping | 0 | 1 | 2 | 3 | 4 |
| 1. Feeling hopeless about the future | 0 | 1 | 2 | 3 | 4 |
| 1. Feeling tense or keyed up | 0 | 1 | 2 | 3 | 4 |
| 1. Feeling everything is an effort | 0 | 1 | 2 | 3 | 4 |
| 1. Spells of terror or panic | 0 | 1 | 2 | 3 | 4 |
| 1. Feeling so restless you couldn’t sit still | 0 | 1 | 2 | 3 | 4 |
| 1. Feelings of worthlessness | 0 | 1 | 2 | 3 | 4 |
| 1. Feeling no interest in things | 0 | 1 | 2 | 3 | 4 |

**Harvard Trauma Questionnaire (HTQ): Traumatic Events (part 1)**

We would like to ask you about your past experiences prior to seeking asylum in Hong Kong. However, you may find some questions upsetting. If so, please feel free not to answer. All answers to the questions will be kept confidential.

Please indicate whether you have experienced any of the following events by marking the box next to each question with YES or NO.

|  | YES | NO |
| --- | --- | --- |
| 1. Lack of shelter (when needed) |  |  |
| 1. Lack of food or water |  |  |
| 1. Ill health without access to medical care |  |  |
| 1. Confiscation or destruction of personal property |  |  |
| 1. Combat situation (e.g. shelling and grenade attacks) |  |  |
| 1. Used as a human shield |  |  |
| 1. Exposure to frequent and unrelenting sniper fire |  |  |
| 1. Forced evacuation under dangerous conditions |  |  |
| 1. Beating to the body |  |  |
| 1. Rape |  |  |
| 1. Other types of sexual abuse or sexual humiliation |  |  |
| 1. Knifing or axing (being injured by knife or axe) |  |  |
| 1. Torture (while in captivity you received deliberate and systematic infliction of physical or mental suffering) |  |  |
| 1. Serious physical injury from combat (e.g., shrapnel, burn, bullet wound, stabbing, etc.) or landmine |  |  |
| 1. Imprisonment |  |  |
| 1. Forced labor (like animal or slave) |  |  |
| 1. Extortion or robbery |  |  |
| 1. Brainwashing |  |  |
| 1. Forced to hide |  |  |
| 1. Kidnapped |  |  |
| 1. Other forced separation from family members |  |  |
| 1. Forced to find and bury bodies |  |  |
| 1. Enforced isolation from others |  |  |
| 1. Present while someone searched for people or things in your home (or the place where you were living) |  |  |
| 1. Forced to sing songs you did not want to sing |  |  |
| 1. Someone was forced to betray you and place you at risk of death or injury |  |  |
| 1. Confined to home because of danger outside |  |  |
| 1. Prevented from burying someone |  |  |
| 1. Forced to desecrate or destroy the bodies or graves of deceased persons |  |  |
| 1. Forced to physically harm family member or friend |  |  |
| 1. Forced to physically harm someone who is not family or friend |  |  |
| 1. Forced to destroy someone else's property or possessions |  |  |
| 1. Forced to betray family member or friend, placing them at risk of death or injury |  |  |
| 1. Forced to betray someone who is not family or friend, placing them at risk of death or injury |  |  |
| 1. Murder or death due to violence of spouse |  |  |
| 1. Murder or death due to violence of son or daughter |  |  |
| 1. Murder or death due to violence of other family member or friend |  |  |
| 1. Disappearance or kidnapping of spouse |  |  |
| 1. Disappearance or kidnapping of son or daughter |  |  |
| 1. Disappearance or kidnapping of other family member or friend |  |  |
| 1. Serious physical injury of family member or friend due to combat situation/ land mine |  |  |
| 1. Witness beatings to head or body |  |  |
| 1. Witness torture |  |  |
| 1. Witness killing or murder |  |  |
| 1. Witness rape or sexual abuse |  |  |
| 1. Any other situation that was very frightening or in which you felt your life was in danger. Please specify all situations that have not been mentioned above. |  |  |

**Harvard Trauma Questionnaire (HTQ): Brain Injury (part 2)**

This section of the questionnaire asks about events you may have experienced that could cause brain injury. For each question, please indicate whether or not you have experienced each event, and if YES, please also indicate whether or not you lost consciousness and for how long. Have you ever experienced…?

|  | Experienced? | | Loss of consciousness? | | If yes, for how long? | |
| --- | --- | --- | --- | --- | --- | --- |
|  | YES | NO | YES | NO | HOURS | MINUTES |
| 1. Beatings to the head |  |  |  |  |  |  |
| 1. Suffocation or Strangulation |  |  |  |  |  |  |
| 1. Near Drowning |  |  |  |  |  |  |
| 1. Injury to the head from a nearby explosion |  |  |  |  |  |  |
| 1. Other types of injury to the head (E.g., shrapnel, bullet wound, stabbing, burns, etc.) |  |  |  |  |  |  |
| 1. Starvation (lack of food for a long period of time) |  |  |  |  |  |  |

If you have experienced starvation (lack of food for a long period of time), please answer the following questions:

1. Please give your normal weight and your starvation weight.

__________________ normal weight

__________________ starvation weight

1. Were you near death due to starvation?

_________ yes

_________ no

**Harvard Trauma Questionnaire (HTQ): PTSD (part 3)**

The following are symptoms people sometimes have after experiencing hurtful or terrifying events in their lives. Please read each statement carefully and decide how much the symptoms bothered you in the past week.

The options are as follows:

**NOT AT ALL, A LITTLE, QUITE A BIT, EXTREMELY**

|  | Not at all | A little | Quite a bit | Extremely |
| --- | --- | --- | --- | --- |
| 1. Recurrent thoughts or memories of the most hurtful or terrifying events |  |  |  |  |
| 1. Feeling as though the event is happening again |  |  |  |  |
| 1. Recurrent nightmares |  |  |  |  |
| 1. Feeling detached or withdrawn from people |  |  |  |  |
| 1. Unable to feel emotions |  |  |  |  |
| 1. Feeling jumpy, easily startled |  |  |  |  |
| 1. Difficulty concentrating |  |  |  |  |
| 1. Trouble sleeping |  |  |  |  |
| 1. Feeling on guard |  |  |  |  |
| 1. Feeling irritable or having outbursts of anger |  |  |  |  |
| 1. Avoiding activities that remind you of the traumatic or hurtful event |  |  |  |  |
| 1. Inability to remember parts of the most hurtful or traumatic events |  |  |  |  |
| 1. Less interest in daily activities |  |  |  |  |
| 1. Feeling as if you don’t have a future |  |  |  |  |
| 1. Avoiding thoughts or feelings associated with the traumatic or hurtful event |  |  |  |  |
| 1. Sudden emotional or physical reaction when reminded of the most hurtful or terrifying events |  |  |  |  |
| 1. Feeling that you have less skills than you had before |  |  |  |  |
| 1. Having difficulty dealing with new situations |  |  |  |  |
| 1. Feeling exhausted |  |  |  |  |
| 1. Bodily pain |  |  |  |  |
| 1. Troubled by physical problem(s) |  |  |  |  |
| 1. Poor memory |  |  |  |  |
| 1. Finding out or being told by other people than you have done something you cannot remember |  |  |  |  |
| 1. Difficulty paying attention |  |  |  |  |
| 1. Feeling as if you are split into two people and one of you is watching what the other is doing |  |  |  |  |
| 1. Feeling unable to make daily plans |  |  |  |  |
| 1. Blaming yourself for things that have happened |  |  |  |  |
| 1. Feeling guilty for having survived |  |  |  |  |
| 1. Without hope |  |  |  |  |
| 1. Feeling ashamed of the hurtful or traumatic events that have happened to you |  |  |  |  |
| 1. Feeling that people do not understand what happened to you |  |  |  |  |
| 1. Feeling that others are hostile to you |  |  |  |  |
| 1. Feeling that you have no one to rely upon |  |  |  |  |
| 1. Feeling someone you trusted betrayed you |  |  |  |  |
| 1. Feeling humiliated by your experience |  |  |  |  |
| 1. Feeling no trust in others |  |  |  |  |
| 1. Feeling powerless to help others |  |  |  |  |
| 1. Spending time thinking why these events happened to you |  |  |  |  |
| 1. Feeling you are the only one who suffered these events |  |  |  |  |
| 1. Feeling a need for revenge |  |  |  |  |

**The Penn State Worry Questionnaire (PSWQ)**

Rate each of the following statements on a scale 1 (“not at all typical of me”) to 5 (“very typical of me”). Please do not leave any items blank

|  | Not at all typical of me |  | | | Very typical of me |
| --- | --- | --- | --- | --- | --- |
| 1. If I do not have enough time to do everything, I do not worry about it. | 1 | 2 | 3 | 4 | 5 |
| 1. My worries overwhelm me. | 1 | 2 | 3 | 4 | 5 |
| 1. I do not tend to worry about things. | 1 | 2 | 3 | 4 | 5 |
| 1. Many situations make me worry. | 1 | 2 | 3 | 4 | 5 |
| 1. I know I should not worry about things, but I just cannot help it. | 1 | 2 | 3 | 4 | 5 |
| 1. When I am under pressure, I worry a lot. | 1 | 2 | 3 | 4 | 5 |
| 1. I am always worrying about something. | 1 | 2 | 3 | 4 | 5 |
| 1. I find it easy to dismiss worrisome thoughts. | 1 | 2 | 3 | 4 | 5 |
| 1. As soon as I finish one task, I start to worry about everything else I have to do. | 1 | 2 | 3 | 4 | 5 |
| 1. I never worry about anything. | 1 | 2 | 3 | 4 | 5 |
| 1. When there is nothing more I can do about a concern, I do not worry about it anymore. | 1 | 2 | 3 | 4 | 5 |
| 1. I have been a worrier all my life. | 1 | 2 | 3 | 4 | 5 |
| 1. I notice that I have been worrying about things. | 1 | 2 | 3 | 4 | 5 |
| 1. Once I start worrying, I cannot stop. | 1 | 2 | 3 | 4 | 5 |
| 1. I worry all the time. | 1 | 2 | 3 | 4 | 5 |
| 1. I worry about projects until they are all done. | 1 | 2 | 3 | 4 | 5 |

**Activities to cope with life difficulties**

|  | None of the time | A little of the time | Quite a lot of the time | Most of the time |
| --- | --- | --- | --- | --- |
| **Spiritual** | | | | |
| 1. Religious or cultural practices |  |  |  |  |
| 1. Visiting spiritual leaders |  |  |  |  |
| 1. Prayer |  |  |  |  |
| 1. Meditation |  |  |  |  |
| **Social** | | | | |
| 1. Engaging in conversations with people |  |  |  |  |
| 1. Going to work |  |  |  |  |
| 1. Engaging in social interactions |  |  |  |  |
| **Distress related behaviours** | | | | |
| 1. Drinking alcohol |  |  |  |  |
| 1. Cigarette |  |  |  |  |
| 1. Aggression |  |  |  |  |
| 1. Overeating |  |  |  |  |
| 1. Visiting a doctor |  |  |  |  |
| 1. Do physical activities |  |  |  |  |
| 1. Sleep or stay in bed |  |  |  |  |

END

Thank you very much
